# An Uncertainty-Aware Bayesian Deep Learning Method for Automatic Identification and Capacitance Estimation of Compensation Capacitors

**DOI:** 10.3390/s26010279

**Published:** 2026-01-02

**Authors:** Tongdian Wang, Pan Wang

**Affiliations:** School of Modern Posts, School of Intelligent Transportation, Nanjing University of Posts and Telecommunications, Nanjing 210003, China; wangtd@njupt.edu.cn

**Keywords:** track circuits, compensation capacitor, Bayesian deep learning, multi-domain signal enhancement, uncertainty-aware diagnosis

## Abstract

This paper addresses the challenges of misclassification and reliability assessment in compensation capacitor detection under strong noise in high-speed railway track circuits. A hierarchical Bayesian deep learning framework is proposed, integrating multi-domain signal enhancement in the time, frequency, and time–frequency (TF) domains with bidirectional long short-term memory (BiLSTM) sequence modeling for robust feature extraction. Bayesian classification and regression based on Monte Carlo (MC) Dropout and stochastic weight averaging Gaussian (SWAG) enable posterior inference, confidence interval estimation, and uncertainty-aware prediction, while a rejection mechanism filters low-confidence outputs. Experiments on 8782 real-world segments from five railway lines show that the proposed method achieves 97.8% state-recognition accuracy, a mean absolute error of 0.084 μF, and an R^2^ of 0.96. It further outperforms threshold-based, convolutional neural network (CNN), and standard BiLSTM models in negative log-likelihood (NLL), expected calibration error (ECE), and overall calibration quality, approaching the theoretical 95% interval coverage. The framework substantially improves robustness, accuracy, and reliability, providing a viable solution for intelligent monitoring and safety assurance of compensation capacitors in track circuits.

## 1. Introduction

The track circuit is a fundamental component of railway signaling systems, supporting train occupancy detection and signal transmission, and thereby serving as a key determinant of operational safety and efficiency [1,2]. Compensation capacitors regulate track impedance, extend transmission distance, and maintain signal quality; the induced voltage waveform therefore reflects both capacitor condition and overall circuit performance [3,4]. Accurate state identification and capacitance estimation are thus essential for ensuring system reliability and enabling intelligent maintenance. Traditional capacitor detection relies heavily on expert interpretation and threshold-based waveform criteria—such as peak decay and pulse-response variations—or on coarse capacitance estimation derived from band-energy changes. Although wavelet TF analysis, empirical mode decomposition, and support vector machines have been explored for track circuit fault detection, these approaches still suffer from limited automation, rigid thresholds, and insufficient robustness under strong noise, structural interference, and operational variability.

In broader industrial monitoring domains, CNNs, autoencoders, and CNN–SVM hybrids demonstrate strong discriminative capability for bearings, motors, and other rotating machinery [5,6,7,8]. Nevertheless, these models produce deterministic outputs and lack mechanisms to quantify predictive confidence—an essential requirement in railway signaling, where decision reliability is as critical as accuracy [9,10]. Bayesian deep learning addresses this limitation by modeling network parameters as random variables and generating predictive distributions through approximate inference. MC provides lightweight posterior sampling and confidence interval estimation, while SWAG yields calibrated Gaussian posterior approximations with strong stability across classification and transfer learning tasks [10,11,12].

Uncertainty-aware deep learning has therefore gained prominence in industrial fault diagnosis, as uncertainty quantification improves interpretability, mitigates model overconfidence, and supports rejection strategies for safety-critical decision processes [13,14,15]. However, research on compensation capacitors continues to rely on threshold-based approaches or conventional deep models, failing to jointly address state classification, capacitance regression, and uncertainty quantification. Capacitor signals are further complicated by random noise, low-frequency drift, and narrowband structural interference, which can introduce spurious peaks and lead to misclassification. Moreover, existing deep learning frameworks lack calibrated confidence intervals, limiting their practical value.

To address these limitations, this paper proposes a Bayesian deep learning framework capable of automatic capacitor state recognition and capacitance estimation while explicitly modeling predictive uncertainty. The framework first performs multi-domain signal enhancement—including operations in the time, frequency, and TF domains—to attenuate noise and strengthen capacitor-related signatures. A BiLSTM network is then employed for sequence modeling, followed by Bayesian classification and regression modules that generate predictive distributions and associated confidence intervals. Uncertainty estimation is further refined through MC Dropout and SWAG, and a rejection mechanism is incorporated to enhance diagnostic reliability in safety-critical environments.

The primary contributions of this study are as follows:

(i) A multi-domain dataset and dual-source annotation strategy integrating manual labeling with field inspection data;
(ii) A multi-domain signal enhancement and feature-fusion framework incorporating peak reinforcement, FD energy reshaping, and combined Short Time Fourier Transform (STFT) processing;
(iii) A hierarchical Bayesian deep learning architecture unifying state classification, capacitance regression, and uncertainty quantification;
(iv) A low-confidence rejection mechanism that improves system trustworthiness by suppressing unreliable inferences.

Extensive experiments on data collected from five operational railway lines demonstrate substantial improvements in state-recognition accuracy, capacitance-estimation precision, and uncertainty calibration over threshold-based and conventional deep learning baselines, confirming the practical effectiveness of the proposed framework for intelligent monitoring and safe operation of track circuit compensation capacitors.

## 2. Related Work

### 2.1. Deep Learning-Based Track Circuit and Railway Infrastructure Monitoring

Deep learning has significantly advanced railway infrastructure monitoring and track circuit safety, becoming central to improving automation and diagnostic reliability. Several surveys summarize its progress in anomaly detection, structural degradation analysis, and surface defect identification. Ji et al. reviewed deep learning methods for rail condition monitoring, noting improvements in detection sensitivity while highlighting challenges in sample scarcity and limited generalization [16]. Di Summa et al. surveyed deep learning applications in infrastructure health monitoring, emphasizing the importance of multi-modal sensing and spatiotemporal feature fusion [17]. At a broader system level, Tang et al. discussed artificial intelligence applications in scheduling, signaling, and maintenance [18], whereas Oh et al. reviewed deep learning solutions for railway safety tasks including collision warning, intrusion detection, and signal equipment monitoring [19].

For track circuits specifically, various deep models have been developed to enhance feature representation and robustness under operational variability. Tao et al. proposed a multi-scale attention network (MSAN) that converts monitoring signals into Gramian Angular Field images and extracts features using multi-scale convolution and attention, outperforming CNNs in multi-class fault diagnosis [20]. To mitigate data scarcity, Na et al. introduced heterogeneous transfer learning using external degradation datasets as source domains to improve diagnostic generalization [21].

Although recent work has progressed toward predictive maintenance, cross-domain adaptation, and multi-source fusion, fine-grained modeling and uncertainty-aware capacitance estimation for key components such as compensation capacitors remain underexplored. A unified framework that maintains robustness under strong noise and varying operating conditions while providing reliable and interpretable outputs is still lacking.

### 2.2. Compensation Capacitor Fault Diagnosis and Health State Prediction

Research on compensation capacitors—critical components of track circuits—has evolved from traditional machine learning to deep learning approaches. Li et al. employed a probabilistic neural network (PNN) for capacitor fault localization by analyzing current waveform alterations, achieving high diagnostic accuracy across fault modes [22]. Gao et al. proposed an enhanced long short-term memory (LSTM) model optimized by Moth–Flame Optimization (MFO-LSTM) for forecasting fault quantities, demonstrating improved accuracy and faster convergence over standard LSTM [23].

For capacitor health prediction, Wang et al. developed an AI-based composite modeling strategy that integrates multiple learners to construct degradation curves and assess health grades using real inspection data [24]. Chen et al. introduced a phase-space–network hybrid diagnostic scheme by embedding detection signals into phase space and constructing single-layer directed visible graphs (SLPVGs), achieving nearly 99% recognition accuracy through topological metrics such as betweenness centrality [25].

These studies contribute to fault localization, short-term fault trend prediction, and health assessment. However, the existing literature remains fragmented across tasks, lacks uncertainty modeling, and depends on limited feature perspectives. A unified framework enabling multi-task learning, robustness under complex conditions, and strict uncertainty quantification is still required.

### 2.3. Bayesian Deep Learning and Uncertainty-Aware Fault Diagnosis

In safety-critical systems, deterministic deep learning models struggle with distribution shifts, complex operational conditions, and limited samples, making uncertainty quantification indispensable for reliable decision-making. Cheliotis et al. reviewed Bayesian and machine-learning-based fault detection approaches, underscoring the importance of probabilistic modeling for risk-aware inference [26]. Zhao et al. summarized robustness and interpretability advances in rolling-element bearing diagnosis and emphasized the necessity of uncertainty estimation [27].

Methodologically, Zhou et al. proposed a probabilistic Bayesian deep learning framework that models parameter uncertainty and input noise for identifying unknown operating conditions [28] and later introduced a Bayesian CNN-based uncertainty-driven diagnostic architecture [29]. Meng et al. developed a Bayesian parallel deep learning system using multi-branch feature extraction to improve recognition stability for wind turbine bearings [30].

From a unified uncertainty modeling perspective, Jalayer et al. compared MC Dropout, Bayesian neural networks, and deep ensembles for analyzing epistemic and aleatoric uncertainty and detecting out-of-distribution samples [31]. Lin et al. incorporated confidence calibration techniques such as temperature scaling into diagnostic frameworks [32], while Li et al. proposed a Bayesian deep learning model with joint prior–posterior calibration for refined uncertainty characterization [33]. The Evidence-Based Optimal Decision Control Model (EODCM) introduced trust metrics and evidence distributions into CNNs to obtain calibrated confidence intervals under high-noise conditions [34]. Siami et al. employed Bayesian decision averaging to fuse predictions and uncertainties from multiple sensor CNNs, enhancing multi-sensor diagnostic robustness.

Despite promising results in bearings, motors, and wind turbine systems, uncertainty-aware deep learning for compensation capacitors remains limited. Existing work lacks capabilities for multi-domain signal modeling, joint state classification and capacitance regression, calibrated uncertainty quantification, and rejection control. Bridging this gap motivates the Bayesian deep learning framework proposed in this study.

## 3. Problem Description and Signal Modeling

### 3.1. Problem Definition

**Figure 1:**
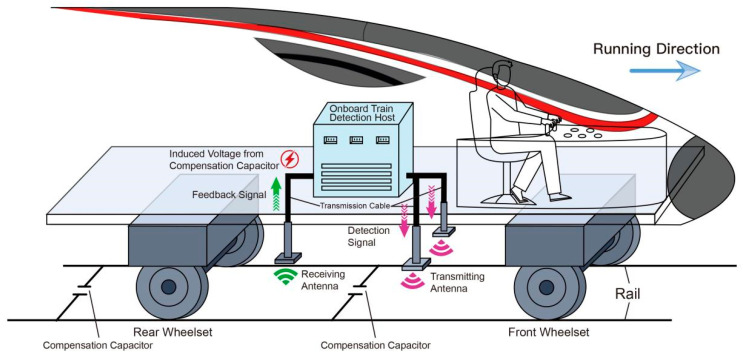
Overall schematic diagram of the track circuit compensation capacitor detection system.

In track circuit detection systems, variations in the compensation capacitor directly modify loop impedance and consequently affect the induced current and received voltage amplitude. Online monitoring is performed using an inspection vehicle equipped with a detection host, transmitting and receiving antennas, and signal transmission cables, as shown in Figure 1. The host generates an alternating current (AC) excitation signal that is delivered to two rectangular transmitting antennas beneath the vehicle, producing an alternating magnetic field in the track circuit. This excitation induces an electromagnetic response whose characteristics depend on the loop impedance.

At the receiver side, the alternating magnetic field generates an electromotive force (EMF) and loop current according to Maxwell–Ampère and Faraday induction principles, with the response magnitude governed by impedance variations. As the vehicle passes over a compensation capacitor, the impedance decreases and then rises, forming a characteristic rise–fall peak pattern in the received voltage. Receiving antennas above the rail centerline capture these responses for waveform analysis. Different capacitor states exhibit distinct amplitude characteristics: prominent peaks for the Normal state, absence of peaks for the Disconnected/Missing state, and attenuated peaks for the Capacitance-Decline state (typically when the amplitude drops to approximately half of the regional average).

Accordingly, compensation capacitor detection involves three core tasks: (i) state recognition, identifying whether the capacitor is present and functional; (ii) capacitance estimation, inferring degradation from peak characteristics; and (iii) confidence evaluation, quantifying predictive uncertainty. Traditional waveform-based techniques rely on manual interpretation, fixed thresholds that degrade under noise and variable operating conditions, and lack uncertainty quantification. Noise and structural interference may also introduce spurious peaks, further increasing the likelihood of misclassification.

To address these limitations, this work proposes a unified framework that integrates physics-based signal modeling with Bayesian uncertainty reasoning to achieve automatic capacitor state recognition and capacitance estimation. By incorporating calibrated uncertainty quantification, the framework enhances robustness and reliability under complex and noise-intensive operating conditions.

### 3.2. Signal Modeling

#### 3.2.1. Time-Domain (TD) Model

During the operation of the inspection vehicle, the AC excitation signal generated by the detection host can be represented as:

(1) $$s(t)=A_{s}cos\left(\omega t+\varphi_{s}\right)$$

where $A_{s}$ is the amplitude of the transmitted signal, ω is the angular frequency, and ϕ_s_ is the initial phase. This signal is transmitted through the cables to drive the two transmitting antennas, which inject a magnetic field into the track circuit. The alternating magnetic field in the closed loop induces an EMF, according to Faraday’s law:

(2) $$e(t)=-\frac{d\Phi (t)}{dt}$$

where Φ(t) is the magnetic flux through the closed track loop. The magnetic flux is determined by the magnetic field strength and the equivalent area:

(3) Φ(t)=B(t)⋅S

where B(t) is the magnetic field strength generated by the transmitting antennas, and *S* denotes the effective flux-coupling area of the receiving loop. The magnetic field generated by the transmitting antenna can be approximated as:

(4) $$B(t)=B_{0}cos\left(\omega t+\varphi_{b}\right)$$

where $B_{0}$ is the magnetic field amplitude, and $\varphi_{b}$ is the magnetic field phase. Therefore, the induced EMF is given by:

(5) $$e(t)=\omega B_{0}Ssin\left(\omega t+\varphi_{b}\right)$$

The track and train wheelset form a closed loop that can be equivalently modeled as a series RLC circuit, with the equivalent impedance given by:

(6) $$Z_{eq}=R+j\left(\omega L-\frac{1}{\omega C}\right)$$

where R is the loop’s equivalent resistance, L is the equivalent inductance, and C is the capacitance of the compensation capacitor. According to Kirchhoff’s voltage law, the induced current is:

(7) $$i(t)=\frac{e_{0}}{\left|Z_{eq}\right|}cos\left(\omega t+\varphi_{i}\right)$$

where $\varphi_{i}$ is the phase shift caused by the impedance. The receiving antenna is located directly above the midpoint of the track. According to Ampère–Maxwell’s law, the induced current generates a secondary magnetic flux in the external space, resulting in a received voltage:

(8) $$\nu_{r}(t)=\frac{N_{r}\mathrm{KL}\omega e_{0}}{\left|Z_{\mathrm{eq}}\right|}\cos\left(\omega t+\varphi_{v}\right)$$

where $N_{r}$ is the number of turns of the receiving antenna, K denotes the electromagnetic coupling coefficient between the transmitting and receiving antennas, and $\varphi_{v}$ is the combined phase at the receiver. The relationship between the received amplitude and the capacitance value is:

(9) $$A_{v}(C)\propto \frac{1}{\sqrt{R^{2}+{\left(\omega L-\frac{1}{\omega C}\right)}^{2}}}$$

Thus, the TD signal model reveals the typical time-dependent behavior of the received voltage as the compensation capacitor enters the loop: as the capacitor gradually enters the closed loop, the received voltage amplitude increases and reaches a peak at a certain point; as the capacitor gradually exits the loop, the amplitude decreases again. If the capacitance value decreases, the peak amplitude weakens proportionally. If the capacitor is disconnected, the peak signal disappears entirely.

#### 3.2.2. Frequency-Domain (FD) Model

The input excitation in the frequency domain can be represented as:

S(ω) = F{s(t)}(10)

where F{·} is the Fourier transform. The frequency spectrum of the loop’s EMF is:

(11) E(ω)=jωΦ(ω)

where Φ(ω) is the frequency spectrum of the magnetic flux. The loop current satisfies:

(12) $$I(\omega )=\frac{E(\omega )}{Z_{eq}(\omega )}$$

Considering the coupling relationship between the receiving antenna and the loop current, the Fourier transform of the received signal is:

(13) $$V_{r}(\omega )=H(\omega )\cdot I(\omega )$$

where H(ω) is the coupling transfer function of the transmit-receive link, including physical parameters such as the receiving antenna area, number of turns, and coupling coefficient. The frequency response function is:

(14) $$H_{eq}(\omega )=K\frac{j\omega }{R+j\left(\omega L-\frac{1}{\omega C}\right)}$$

The amplitude–frequency response is:

(15) $$\left|H_{eq}(\omega )\right|=K\frac{\omega }{\sqrt{R^{2}+{\left(\omega L-\frac{1}{\omega C}\right)}^{2}}}$$

The state of the compensation capacitor influences the following spectral features:

- Normal: A clear resonance peak appears at ω ≈ $1/\sqrt{LC_{0}}$.
- Capacitance Drop: The peak amplitude decreases, and the main peak shifts to the right.
- Disconnected Fault: The resonance characteristics disappear, and the spectrum becomes flat.

#### 3.2.3. Time–Frequency Domain Model

When the compensation capacitor enters the closed loop, the received voltage exhibits a typical narrowband amplitude–phase modulated structure, which can be represented as:

(16) $$y(t)=A(t)cos\left(\omega_{0}t+\phi (t)\right)+\varepsilon (t)$$

where A(t) is the slowly time-varying amplitude envelope, ϕ(t) is the slowly time-varying phase, ω_0_ is the system’s working angular frequency, and ε(t) is the additive noise and interference (including Gaussian/colored noise and resonance terms). The capacitance value of the compensation capacitor influences the loop impedance, so the amplitude envelope satisfies:

(17) $$A(t)\propto \frac{1}{\sqrt{R^{2}+{\left(\omega_{0}L-\frac{1}{\omega_{0}C(t)}\right)}^{2}}}$$

This reflects the physical law of the capacitor’s “enter–center–leave” process, where the amplitude first increases and then decreases.

(1) STFT Time–Frequency Representation

To capture the transient energy changes caused by the compensation capacitor, we first perform an STFT on the signal:

(18) $$X_{y}(t,\omega )=\int y(\tau )h^{*}(\tau -t)e^{-j\omega \tau }d\tau$$

Its time–frequency energy distribution is:

(19) $$S_{y}(t,\omega )={\left|X_{y}(t,\omega )\right|}^{2}$$

This representation visually reflects the local energy enhancement at the position where the compensation capacitor enters.

(2) Continuous Wavelet Transform (CWT) Representation

The CWT is:

(20) $$W_{y}(a,b)=\frac{1}{a}\int y(t)\psi^{*}\left(\frac{t-b}{a}\right)dt$$

The resulting scale-time plot ${\left|W_{y}(a,b)\right|}^{2}$ highlights the impact of low-frequency disturbances and narrowband structural interference on the signal.

(3) Energy Ridge and Brightband Features

The time–frequency representation of the compensation capacitor’s position typically shows an “energy ridge” along the time axis. The central frequency of the ridge can be obtained by:

(21) $$\omega *(t)\;=\;\arg\underset{\omega }{\max}S_{y}(t,\omega )$$

and its local energy is defined as:

(22) $$E(t)=\int_{B(\omega *(t))}\;S_{y}(t,\omega )d\omega$$

where B(ω*(t)) represents the bandwidth of the ridge neighborhood.

(4) Mapping Relationship Between Brightband Energy and Capacitance Value

The brightband energy is monotonically related to the capacitance value and can be approximated as:

(23) $$E(t)\approx \beta \cdot \frac{1}{R^{2}+{\left(\omega_{0}L-\frac{1}{\omega_{0}C(t)}\right)}^{2}}+\xi (t)$$

where β is the normalization coefficient and ξ(t) denotes a zero-mean noise term that accounts for measurement noise, environmental interference, and residual modeling errors.

### 3.3. Interference and Noise Modeling

In actual track circuit detection environments, the received signal not only includes the valid response of the compensation capacitor s_c_(t) but also superimposes multiple sources of noise and interference. To establish a unified analysis framework, we categorize these into three typical components: random noise, low-frequency disturbances, and structural narrowband interference.

A. Random Noise

Environmental electromagnetic fields and thermal noise from detection equipment jointly contribute to random noise $n_{g}(t)$, which is typically approximated by zero-mean Gaussian noise:

(24) $$n_{g}(t)∼N\left(0,\sigma^{2}\right)$$

This type of noise manifests as random jitter in the time domain and forms a stable noise floor in the frequency domain, which reduces the stability of peak detection.

B. Low-Frequency Disturbance

Track attenuation, power supply baseline drift, and other factors introduce slowly varying low-frequency components:

(25) $$n_{b}(t)=A_{b}\cos\left(\omega_{b}t+\varphi_{b}\right),\;\omega_{b}\ll \omega_{0}$$

where $\omega_{0}$ is the system’s working angular frequency. Low-frequency disturbances cause slow baseline drift, making it harder to stably identify the compensation capacitor’s envelope.

C. Structural Narrowband Interference

In structural scenarios such as bridges and tunnels, electromagnetic resonance often occurs, adding narrowband components near the working frequency $\omega_{0}$ to the signal:

(26) $$n_{r}(t)=\sum_{k=1}^{K}\;A_{k}cos\left(\omega_{k}t+\varphi_{k}\right),\omega_{k}\approx \omega_{0}$$

This type of interference poses the greatest risk because its frequency is very close to the target frequency band of the compensation capacitor, creating false peaks at non-compensation positions.

D. Comprehensive Noise Model

By integrating the three types of interference, the received signal can be represented as:

(27) $$y(t)=s_{c}(t)+n_{g}(t)+n_{b}(t)+n_{r}(t)$$

where $s_{c}(t)$ is the valid signal of the compensation capacitor. To measure the interference strength, we introduce the signal-to-noise ratio over the target frequency band Ω_c_, where Ω_c_ denotes the operating frequency band centered around the excitation frequency (5–6 kHz in this study):

(28) $$SNR=\frac{\int_{\Omega_{c}}\;S_{c}(\omega )d\omega }{\int_{\Omega_{c}}\;\left(S_{n_{g}}(\omega )+S_{n_{b}}(\omega )+S_{n_{r}}(\omega )\right)d\omega }$$

If the SNR is too low, traditional peak-based recognition methods are highly prone to misjudgment.

As shown in the above analysis, random noise leads to a decrease in signal stability, low-frequency disturbances cause envelope and baseline shifts, and structural narrowband interference generates false peaks within the target frequency band. These three types of interference collectively reduce the separability of the compensation capacitor signal in the time, frequency, and time–frequency domains, which is the fundamental reason for the failure of traditional threshold-based methods in complex working conditions. Therefore, subsequent multi-domain signal enhancement and Bayesian uncertainty modeling are necessary to achieve robust suppression of noise and interference and reliable identification.

## 4. Methodology

Building on the clarified electromagnetic mechanisms, signal patterns, and interference characteristics of compensation capacitors, this section introduces a hierarchical Bayesian deep learning framework that unifies state identification, capacitance regression, and uncertainty quantification. The framework establishes an end-to-end signal understanding and diagnostic system designed to overcome the limited robustness, rigid rule dependence, and absence of credibility estimation inherent in traditional threshold-based approaches under complex operating conditions.

**Figure 2:**
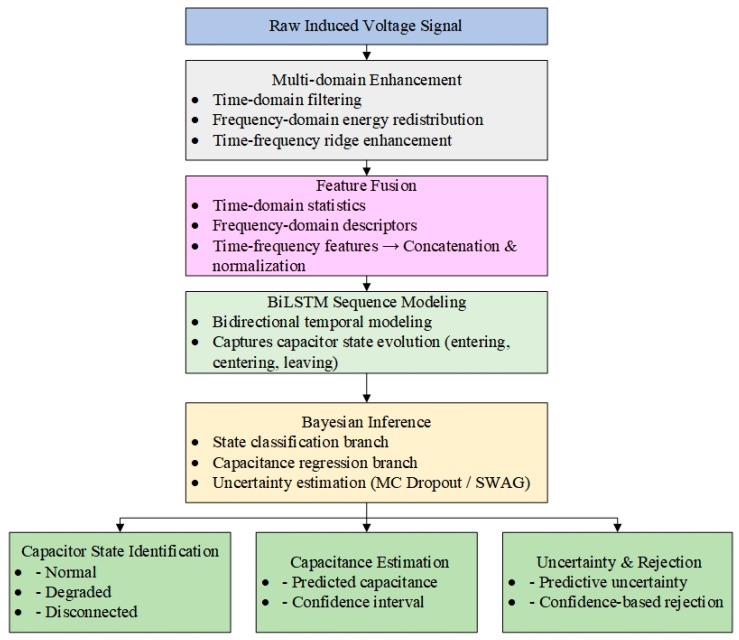
Overall Model Architecture.

The framework in Figure 2 comprises four modules.

(1) Multi-domain Enhancement: TD, FD, and time–FD operations—including bandpass filtering, spectral energy redistribution, and brightband reinforcement—suppress noise and enhance capacitor-related signatures.
(2) Feature Fusion: TD statistics, spectral descriptors, and TF structures are extracted and normalized into a unified multi-view feature vector.
(3) BiLSTM Modeling: A BiLSTM captures the bidirectional temporal evolution of capacitor responses during vehicle approach, passage, and departure.
(4) Bayesian Inference: A Bayesian dual-branch head performs state classification and capacitance regression with uncertainty estimates. MC Dropout and SWAG yield posterior means, variances, and confidence-based rejection.

### 4.1. Multi-Domain Signal Enhancement

In the track environment, the raw received signal contains both the valid components of the compensation capacitor and superimposed random noise, low-frequency drift, and structural narrowband interference (as discussed in Section 3.3). Directly inputting this signal into the model can lead to false peaks and inaccurate capacitance estimates. To enhance signal separability, this study designs a multi-domain enhancement module in the time, frequency, and time–frequency domains.

A. TD Enhancement

The raw signal is expressed as:

(29) $$y(t)=s_{c}(t)+n(t),\;n(t)=n_{g}(t)+n_{b}(t)+n_{r}\left(t\right)$$

where $s_{c}\left(t\right)$ represents the valid component of the compensation capacitor signal, and n(t) represents the noise and interference.

1. Bandpass Filtering

To suppress low-frequency drift and high-frequency noise, the signal is subjected to bandpass filtering:

(30) $$y_{b}(t)=\int_{-\infty }^{\infty }\;y(\tau )h_{\mathrm{bp}}(t-\tau )d\tau$$

Its frequency response satisfies:

(31) $$H_{bp}(\omega )=\left\{\begin{array}{ll} 1, & \omega_{l}\leq \omega \leq \omega_{h}\\ 0, & \;\mathrm{otherwise}\; \end{array}\right.$$

where $\omega_{l}$ and $\omega_{h}$ are the lower and upper limits of the operating frequency band.

In implementation, a 4th-order Butterworth bandpass filter is adopted for the target frequency band (5–6 kHz or 5.6–6.0 kHz, depending on the experiment), and zero-phase forward–backward filtering is applied to avoid phase distortion.

2. Sliding Normalization

To reduce amplitude differences at different positions, the bandpass-filtered signal is locally normalized:

(32) $$y_{n}(t)=\frac{y_{b}(t)-\mu_{w}(t)}{\sigma_{w}(t)+\epsilon }$$

where $\mu_{w}\left(t\right)$ and $\sigma_{w}\left(t\right)$ are the sliding window mean and standard deviation, and ϵ is a stability term.

3. Differential Enhancement

To highlight the envelope fluctuations caused by the capacitor entering/leaving, first-order differences are computed:

(33) $$d(t)=y_{n}\left(t\right)-y_{n}(t-\Delta t)$$

B. FD Enhancement

The compensation capacitor response has energy concentration in the operating frequency band, so FD enhancement is applied.

1. Fourier Transform

The Fourier transform of the signal is:

(34) $$Y(k)=\sum_{n=0}^{N-1}\;y_{n}\left(nT_{s}\right)e^{-j2\pi \mathrm{kn}/N},\;k=0,\ldots ,N-1$$

where $T_{s}$ is the sampling interval.

2. FD Smoothing and Noise Floor Suppression

Local smoothing of the spectrum is performed:

(35) $$\tilde{Y}(\omega )=\frac{1}{2M+1}\sum_{m=-M}^{M}\;Y(\omega +m)$$

The enhanced spectrum is obtained by subtracting the estimated noise floor η(ω):

(36) $$Y_{f}(\omega )=\max\{\tilde{Y}(\omega )-\eta (\omega ),0\}$$

C. Time–frequency Domain Enhancement

To capture transient patterns in the dynamic process of the compensation capacitor, time–frequency analysis is introduced.

1. STFT

The STFT of the signal is:

(37) $$X(t,\omega )=\int_{-\infty }^{\infty }\;y(\tau )h*(\tau -t)e^{-j\omega \tau }d\tau$$

The spectrogram is:

(38) $$S(t,\omega )=|X(t,\omega ){|}^{2}$$

2. Energy Ridge Extraction

The target frequency ridge is obtained by maximizing the criterion:

(39) $$\omega *(t)=\arg\max_{\omega \in \Omega_{c}}\;S(t,\omega )$$

The corresponding ridge energy is:

(40) $$E_{r}(t)=\int_{\omega \in B\left(\omega^{*}(t)\right)}\;S(t,\omega )d\omega$$

3. Synchronous Squeezing Transform (SST)

To improve time–frequency energy focusing, the synchronous squeezing transform is introduced:

(41) $$T_{\mathrm{SST}}(t,\omega )=\int_{0}^{\infty }\;W_{y}\left(a,t\right)a^{-3/2}\delta (\omega -\omega (t,a))\mathrm{da}$$

where $W_{y}\left(a,t\right)$ is the CWT, and ω(t,a) is the instantaneous frequency estimate. SST reallocates energy to improve ridge focusing and further suppress false peaks.

D. Combined Enhancement Output

Finally, the results of the three domains are fused with weighted combination:

(42) $$F(t)=\lambda_{1}y_{n}(t)+\lambda_{2}F_{f}(t)+\lambda_{3}F_{\mathrm{tf}}\left(t\right)$$

where $y_{n}(t)$ is the TD enhancement result, $F_{f}\left(t\right)$ is the inverse FD result, and $F_{\mathrm{tf}}\left(t\right)$ is the time–frequency ridge energy trajectory. The fusion weights are denoted as λ1, λ2, λ3, which are non-negative coefficients satisfying λ1 + λ2 + λ3 = 1 and are tuned on the validation set.

This multi-domain enhancement strategy simultaneously suppresses low-frequency drift, attenuates non-target frequency noise, and enhances the local time–frequency brightband features of the compensation capacitor, providing a more robust and discriminative input for subsequent deep learning models.

### 4.2. Feature Extraction and Fusion

After multi-domain signal enhancement, it remains essential to construct discriminative features that reflect the compensation capacitor’s state and capacitance variations. To this end, features are extracted from the time, frequency, and TF domains, followed by a fusion mechanism to form a unified representation that improves robustness across varying operating conditions.

A. TD Features

Given the enhanced signal sequence $\left\{y(n\right){\}}_{n=1}^{N}$, the following statistical and envelope-related features are extracted:

(1) Root Mean Square (RMS)

(43) $$f_{\mathrm{RMS}}=\sqrt{\frac{1}{N}\sum_{n=1}^{N}\;\;y^{2}\left(n\right)}$$

(2) Kurtosis

(44) $$f_{\mathrm{KU}}=\frac{\frac{1}{N}\sum_{n=1}^{N}\;(y(n)-\mu {)}^{4}}{\sigma^{4}}$$

where μ and σ denote the mean and standard deviation.

(3) Envelope Energy

Using the Hilbert transform, the envelope e(n) is obtained, and the envelope energy is defined as:

(45) $$f_{\mathrm{EE}}=\sum_{n=1}^{N}\;e^{2}\left(n\right)$$

TD features capture amplitude trends and peak structures as the capacitor enters and exits the loop.

B. FD Features

Applying the discrete Fourier transform (DFT) to y(n) yields the spectrum Y(k), k = 0, …, N − 1. Two key features are derived:

(1) Band Energy Ratio (BER)

(46) $$f_{BER}=\frac{\sum_{k\in \Omega_{c}}\;|Y(k){|}^{2}}{\sum_{k=0}^{N-1}\;|Y(k){|}^{2}}$$

where Ω_c_ is the operating frequency band. BER reflects the energy concentration around the compensation capacitor’s response.

(2) Spectral Entropy (SE)

Defining normalized spectral power p(k), the entropy is:

(47) $$f_{\mathrm{SE}}=-\sum_{k=0}^{N-1}\;p(k)\mathrm{logp}(k)$$

Spectral entropy characterizes how concentrated or dispersed the spectrum is, useful for distinguishing normal responses from degraded capacitors.

C. TF Features

Using the STFT spectrogram S(t,ω), the following features are extracted:

(1) Ridge Energy (RE)

The energy ridge within Ω_c_ is defined as:

(48) $$\omega *(t)=\arg\max_{\omega \in \Omega_{c}}\;S(t,\omega )$$

(49) $$f_{\mathrm{RE}}=\sum_{t}\;S\left(t,\omega *\left(t\right)\right)$$

This measures the strength of the characteristic “brightband” associated with capacitor response.

(2) TF Texture Features

Viewing the spectrogram as a grayscale image I(t,ω), texture descriptors are obtained from the gray-level co-occurrence matrix P(i,j):

(50) $$f_{\mathrm{CON}}=\sum_{i,j}\;(i-j{)}^{2}P(i,j),\;f_{\mathrm{ENG}}=\sum_{i,j}\;P(i,j{)}^{2}$$

These features quantify the shape and texture of the brightband, offering robustness under heavy noise.

D. Feature Fusion

All extracted features are concatenated into a unified vector:

(51) $$f={\left[f_{RMS},f_{PF},f_{KU},f_{EE},f_{MFE},f_{BER},f_{SE},f_{TFE},f_{RE},f_{CON},f_{ENG}\right]}^{T}$$

After normalization, the model input matrix becomes:

(52) $$F={\left[f_{1},f_{2},\ldots ,f_{d}\right]}^{T}$$

The fused representation integrates waveform patterns, spectral structures, and TF textures, providing a multi-perspective and robust feature space for subsequent BiLSTM modeling and Bayesian inference.

### 4.3. Sequence Modeling and Bayesian Inference

Once the fused multi-domain features are obtained, the temporal evolution of the capacitor response—characterized by clear sequential dependencies and localized peaks—must be captured. A sequence model is employed, and Bayesian inference is incorporated to quantify predictive uncertainty.

A. Sequence Modeling

Let the feature sequence be:

(53) $$F=\left\{f_{1},f_{2},\ldots ,f_{T}\right\},\;f_{t}\in R^{d}$$

To capture temporal dependencies, a BiLSTM is applied:

(54) $${\vec{h}}_{t}={\mathrm{LSTM}}_{f}(f_{t},{\vec{h}}_{t-1}),{\vec{h}}_{t}={\mathrm{LSTM}}_{b}(f_{t},{\vec{h}}_{t+1})$$

The combined hidden state is:

(55) $$h_{t}=[{\vec{h}}_{t};{\vec{h}}_{t}]\in R^{2h}$$

A sequence-level representation is then obtained via pooling:

(56) $$z=Pooling\left(\left\{h_{1},h_{2},\ldots ,h_{T}\right\}\right)$$

This structure captures both transient effects and global temporal trends as the capacitor enters and leaves the track circuit.

B. Bayesian Classification and Regression

Using the embedding vector **z**:

(1) State Classification

(57) $$p\left(y=c_{k}|z,W_{c}\right)=\mathrm{Softmax}\left(W_{c}z+b_{c}\right)$$

(2) Capacitance Regression

(58) $$\overset{ˆ}{C}=W_{r}z+b_{r}$$

with mean squared error loss:

(59) $$L_{\mathrm{reg}}=\frac{1}{N}\sum_{i=1}^{N}\;{\left({\overset{ˆ}{C}}_{i}-C_{i}\right)}^{2}$$

C. Bayesian Inference Mechanism

Model weights are treated as random variables:

(60) W~q(W|θ)

(1) Monte Carlo Dropout

Running dropout during both training and inference yields multiple stochastic predictions ${\left\{y^{\left(m\right)}\right\}}_{m=1}^{M}$:

(61) $$\overset{-}{y}=\frac{1}{M}\sum_{m=1}^{M}y^{\left(m\right)}$$

(62) $$Var[y]\approx \frac{1}{M}\sum_{m=1}^{M}\;{\left(y^{\left(m\right)}-\bar{y}\right)}^{2}$$

In implementation, dropout is applied to the BiLSTM output representations and the subsequent fully connected layers. During inference, multiple stochastic forward passes are performed to approximate the predictive posterior distribution and quantify model uncertainty.

(2) SWAG

A Gaussian posterior approximation is constructed as:

(63) $$q(W)\approx N\left(W_{\mathrm{SWA}},\Sigma \right)$$

Sampling from this distribution provides stable uncertainty estimates.

D. Multi-task Joint Loss

The final training objective combines classification, regression, and Bayesian regularization:

(64) $$L=\lambda_{c}L_{\mathrm{cls}}+\lambda_{r}L_{\mathrm{reg}}+\lambda_{\mathrm{kl}}D_{\mathrm{KL}}\left(q(W|\theta )\|p(W)\right)$$

This ensures balanced optimization of state identification, capacitance estimation, and uncertainty calibration.

### 4.4. Uncertainty Modeling and Confidence-Based Rejection

In safety-critical railway signaling scenarios, providing only state classification and capacitance estimation is insufficient for reliable decision-making. The model must also quantify predictive uncertainty so that unreliable outputs can be automatically rejected. This section develops predictive uncertainty modeling and confidence-based rejection mechanisms within the Bayesian deep learning framework.

A. Predictive Uncertainty Modeling

Bayesian models generally represent two types of uncertainty:

(1) Aleatoric uncertainty (data uncertainty)

Caused by noise, interference, and varying operating conditions, it is modeled through conditional variance:

(65) $$p(y|z,W)=N\left(\mu (z),\sigma^{2}(z)\right)$$

where μ(z) is the predicted mean and $\sigma^{2}\left(z\right)$ is the network-estimated conditional variance.

(2) Epistemic uncertainty (model uncertainty)

Arising from limited training data and model insufficiency, it is estimated via multiple forward passes using MC Dropout or SWAG:

(66) $${\left\{y^{\left(m\right)}\right\}}_{m=1}^{M},\mu_{p}=\frac{1}{M}\sum_{m=1}^{M}\;y^{\left(m\right)},\sigma_{p}^{2}=\frac{1}{M}\sum_{m=1}^{M}\;{\left(y^{\left(m\right)}-\mu_{p}\right)}^{2}$$

(3) Total predictive uncertainty

Aleatoric and epistemic uncertainties are approximately additive:

(67) $$\sigma_{\mathrm{tot}}^{2}=\sigma^{2}(z)+\sigma_{p}^{2}$$

B. Confidence Measurement

(1) Classification confidence

Classification confidence is defined as the maximum softmax probability:

(68) $$\gamma_{c}={max}_{k\in C}\;p\left(y_{c}=k|z\right)$$

(2) Regression confidence

The model outputs a predictive interval for capacitance estimation:

(69) $$C\pm \kappa \sigma_{\mathrm{tot}}$$

where κ = 1.96 corresponds to a 95% confidence interval.

Regression confidence is then defined as:

(70) $$\gamma_{r}=\exp\left(-\frac{\sigma_{\mathrm{tot}\;}}{\sigma_{\mathrm{ref}\;}}\right)$$

where $\sigma_{\mathrm{ref}\;}$ is a normalization factor.

C. Confidence-based Rejection Mechanism

To avoid unsafe decisions, the system actively rejects predictions with low confidence. The rejection rule is:

(71) $$\overset{ˆ}{y}=\left\{\begin{array}{ll} {arg}_{{max}_{k}\;}p\left(y_{c}=k|z\right), & \;\mathrm{if}\;\gamma_{c}\geq \tau_{c},\\ \;\mathrm{Reject},\; & \;\mathrm{if}\;\gamma_{c}<\tau_{c}, \end{array}\right.,\overset{ˆ}{C}=\left\{\begin{array}{ll} \mu_{p}, & \;\mathrm{if}\;\gamma_{r}\geq \tau_{r},\\ \;\mathrm{Reject},\; & \;\mathrm{if}\;\gamma_{r}<\tau_{r}, \end{array}\right.$$

where $\tau_{c}$ and $\tau_{r}$ are the confidence thresholds for classification and regression, respectively, which are determined on a validation set to balance predictive performance and rejection behavior.

By jointly modeling aleatoric and epistemic uncertainties, the framework not only outputs capacitor states and capacitance estimates but also quantifies their reliability. The rejection mechanism further ensures that, under strong noise, structural interference, or abnormal operating conditions, the system can proactively avoid unsafe decisions—providing robust and trustworthy diagnostics for track circuit monitoring.

## 5. Experiments and Results

### 5.1. Experimental Setup and Data Sources

Real-world signals were collected by an in-service inspection vehicle operating across multiple railway lines. The detection system consists of a host controller, transmitting and receiving antennas, and signal transmission cables. During operation, the host injects an excitation signal into the track circuit while the receiving antennas acquire the corresponding electromagnetic responses in binary form. After export, TD preprocessing was performed according to the sampling rate to generate voltage waveforms and peak sequences. Continuous data streams were subsequently segmented by line topology and mileage to construct individual waveform samples.

A hybrid labeling scheme integrating manual waveform inspection with field maintenance records was employed to obtain reliable annotations. Technicians first examined waveform peaks and, using the known number and characteristic spacing of capacitors within each segment, marked their approximate positions. Post-run maintenance reports documenting the actual physical states of each capacitor—Normal, Degraded, or Missing—were then used as the verified ground-truth labels for model training and evaluation.

Across five railway lines, a total of 8782 valid sections were collected using the detection vehicle. After section-level segmentation, 2334, 1875, 2034, 894, and 1645 sections were obtained from the respective lines.

Each section was labeled into one of three capacitor states, namely Normal, Degraded (Capacitance Drop), and Disconnected (Open Circuit). By aggregating the labeled section-level data from all lines, the complete experimental dataset used in this study was constructed. The detailed dataset statistics and class distribution are summarized in Table 1.

Overall, the dataset exhibits a representative distribution of real-world operating conditions and capacitor degradation modes, providing a reliable foundation for evaluating the proposed Bayesian deep learning framework.

### 5.2. Baseline Methods and Evaluation Metrics

To comprehensively validate the proposed hierarchical Bayesian deep learning framework, we compare it with representative baseline methods covering rule-based detection, conventional deep learning, and uncertainty-aware modeling.

A. Baseline Methods

1. Threshold-based Method:

A classical rule-based approach in which degraded capacitors are identified when peak amplitude falls below a predefined ratio of the local segment mean, and missing capacitors are detected when no peak is present. Its performance degrades severely under strong noise or structural interference.

2. CNN-based Model:

A 1-D CNN applied to enhanced TD signals to learn local patterns. Although effective for local feature extraction, it cannot capture long-range temporal dependencies critical for modeling capacitor dynamics.

3. LSTM-based Model:

A unidirectional LSTM used for state classification and capacitance regression. While capable of modeling temporal evolution, it lacks uncertainty estimation—an essential component for safety-critical railway applications.

4. Proposed Hierarchical Bayesian Deep Learning Framework:

Integrates multi-domain enhancement, multi-feature fusion, BiLSTM sequence modeling, Bayesian classification/regression, and confidence-based rejection. It jointly performs state recognition, capacitance estimation, and uncertainty quantification, substantially improving robustness and safety.

B. Evaluation Metrics

Model performance is assessed across three dimensions: classification, regression, and uncertainty quality. Table 2 summarizes the metrics and their purposes.

Accuracy, Precision, Recall, and F1-score jointly evaluate the reliability and completeness of state recognition, while MAE and RMSE assess capacitance regression accuracy and robustness under varying conditions. For uncertainty evaluation, NLL, ECE, and RR measure predictive credibility, calibration quality, and safety-oriented rejection behavior. Together, these metrics constitute a comprehensive evaluation framework that rigorously validates the superiority of the proposed method under complex noise and diverse operational scenarios.

### 5.3. Effectiveness of Multi-Domain Signal Enhancement

To evaluate the impact of the proposed multi-domain enhancement strategy under complex noise conditions, this section compares the raw and enhanced signals in the time, frequency, and TF domains, and analyzes their contributions to capacitor identification.

A. TD Enhancement

**Figure 3:**
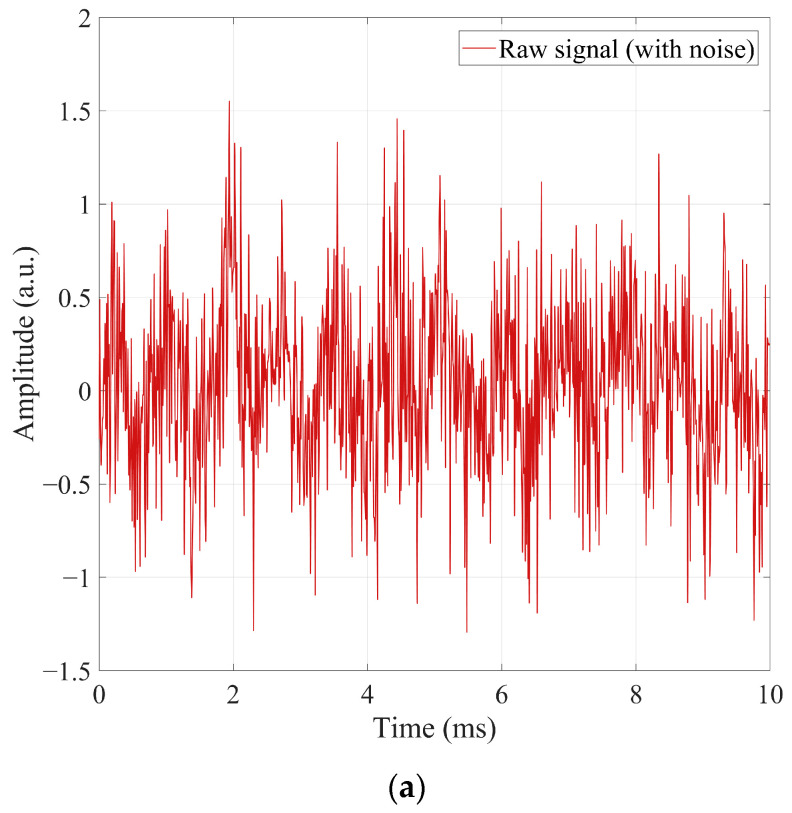

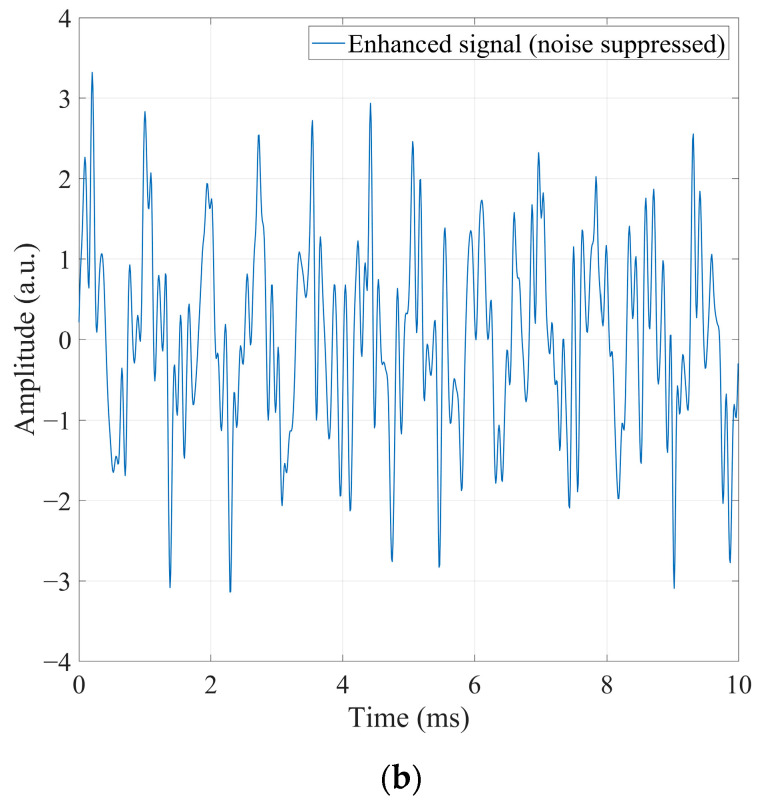
Comparison of TD signals before and after enhancement. (**a**) Raw signal with noise and environmental interference. (**b**) Enhanced signal after multi-domain processing.

Figure 3 illustrates the improvements achieved through TD enhancement under strong noise. As shown in Figure 3a, the raw signal suffers from high-frequency jitter, low-frequency drift, and amplitude fluctuations introduced by random noise, power-frequency interference, and track-induced baseline drift. These distortions obscure the characteristic “enter–center–leave” amplitude pattern of the compensation capacitor, and the overlap between true and false peaks renders traditional threshold-based detection unreliable.

To enhance separability, bandpass filtering, sliding normalization, and differential enhancement are applied. The enhanced signal in Figure 3b shows that:

- High-frequency jitter is effectively suppressed;
- The envelope variation caused by capacitor movement becomes more prominent;
- Low-frequency drift is removed, stabilizing the baseline near zero;
- False peaks are largely eliminated, while true peaks become more distinguishable.

The enhancement quality is quantified using the peak signal-to-noise ratio:

(72) $$PSNR=10\cdot {log}_{10}\left(\frac{max\left(y^{2}(t)\right)}{\sigma_{n}^{2}}\right)$$

where y(t) is the signal and $\sigma_{n}^{2}$ is the noise variance. Experiments show that the enhancement increases PSNR by 6–10 dB, indicating significantly improved stability.

Overall, the TD module effectively restores key amplitude structures and greatly improves the discriminability required for subsequent feature extraction and deep modeling.

B. FD Enhancement

**Figure 4:**
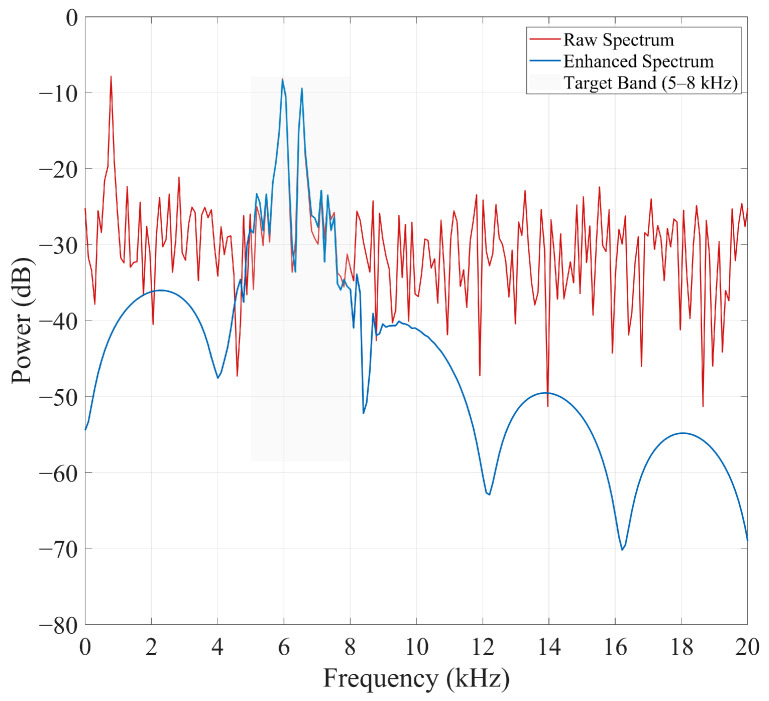
FD comparison before and after enhancement.

Figure 4 shows the power spectra of the raw and enhanced signals: the raw spectrum contains wideband noise with large fluctuations that nearly bury the capacitor-induced response in the 5–6 kHz target band, whereas the enhanced spectrum exhibits strong suppression of wideband noise (20–40 dB), substantial elimination of narrowband structural interference, and a sharp, concentrated peak in the target band that markedly improves detectability.

Energy concentration in the target band is quantified by the BER:

(73) $$BER=\frac{\sum_{f\in \Omega_{c}}\;S(f)}{\sum_{f\in \Omega }\;S(f)}$$

Results show a 15–22% increase in BER after enhancement, demonstrating stronger spectral contrast and improved representation of capacitor-related frequency components.

Thus, FD enhancement effectively suppresses irrelevant noise while retaining and emphasizing physically meaningful resonance features.

C. TF Enhancement

**Figure 5:**
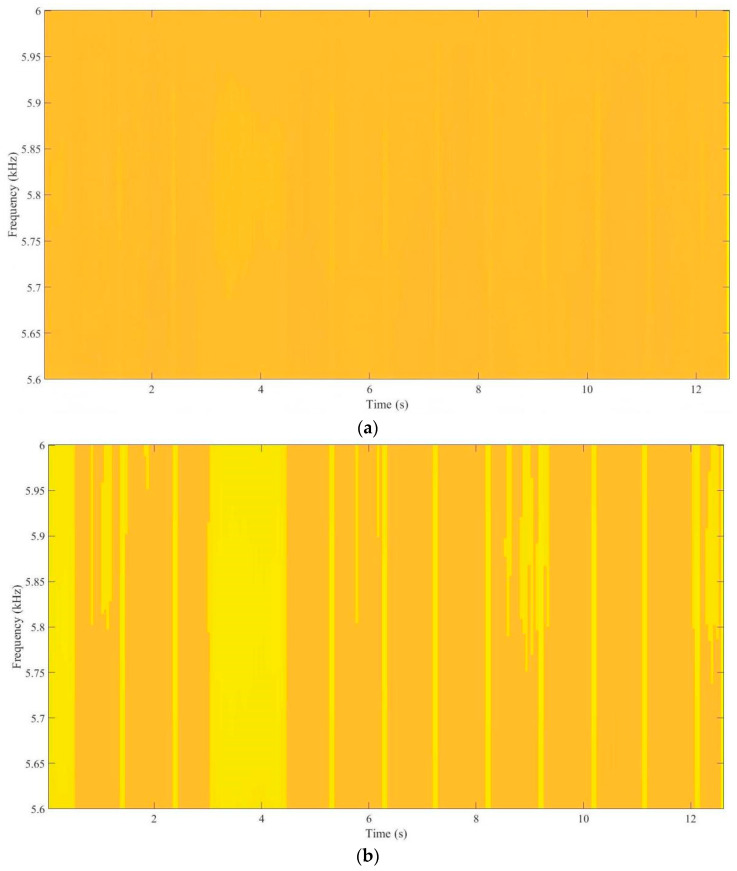
Comparison of TF representations before and after enhancement. (**a**) Raw STFT TF map. (**b**) Enhanced STFT TF map.

Figure 5 evaluates the recovery of transient capacitor-induced patterns via STFT. In Figure 5a, the raw spectrogram is dominated by broadband noise, lacking visible structure in the 5.6–6.0 kHz band. No energy ridges or bright bands can be identified, and the transient variations associated with the capacitor movement are completely obscured.

In contrast, the enhanced spectrogram in Figure 5b shows:

- Distinct high-energy bands appearing periodically within the target frequency range;
- Clear structural patterns replacing the amorphous noise background;
- Well-defined boundaries corresponding to the capacitor’s dynamic movement;
- Significantly reduced noise-induced artifacts.

The enhancement effectiveness is quantified using the Energy Concentration Ratio:

(74) $$ECR=\frac{\sum_{(\tau ,\omega )\in \Lambda }\;\;|Y(\tau ,\omega ){|}^{2}}{\sum_{\tau ,\omega }\;\;|Y(\tau ,\omega ){|}^{2}}$$

where Λ denotes the bright-band region. The ECR improves by approximately 18%, demonstrating enhanced energy focus and clearer extraction of capacitor dynamics.

Across all three domains, the multi-domain enhancement strategy significantly improves peak visibility, suppresses noise, and strengthens physically meaningful signal structures, forming a solid foundation for subsequent learning and inference.

### 5.4. Feature Extraction and Fusion Performance

To evaluate the effectiveness of the proposed multi-domain feature extraction and fusion strategy, two analyses are performed: (1) a quantitative assessment of the discriminative power and stability of individual feature categories, and (2) an ablation study examining the contribution of each feature type and fusion configuration to overall performance.

#### 5.4.1. Discriminative Feature Analysis

**Figure 6:**
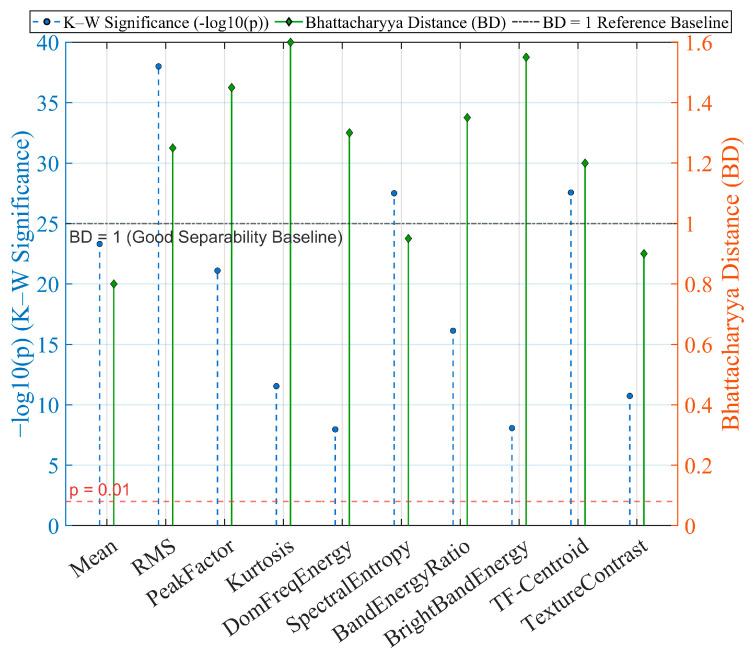
Kruskal–Wallis Significance Test and Bhattacharyya Distance Analysis.

Figure 6 presents the significance and class-separability analysis of TD, FD, and TF features using the Kruskal–Wallis (K–W) test and Bhattacharyya Distance (BD). As described in Section 4.2, the three feature groups include: (i) TD features such as mean, RMS, peak factor, and kurtosis, which capture amplitude evolution and peak structure; (ii) FD features such as dominant-frequency energy, spectral entropy, and band-energy ratio, which reflect spectral changes under capacitor degradation; and (iii) TF features, including ridge intensity, TF centroid, and STFT/CWT-based texture descriptors, which characterize transient patterns during the entering–centering–leaving process.

K–W significance analysis (−log10(p)) shows that TD features—especially RMS and peak factor—exhibit the strongest discrimination across capacitor states, while dominant-energy and BER remain the most informative FD descriptors. TF features contribute complementary information but exhibit weaker significance under certain conditions. BD results further confirm that TD features provide the highest separability, with RMS and peak factor yielding the largest BD values. FD features also form effective class boundaries, whereas some TF descriptors show reduced separability between degraded and disconnected states.

Overall, TD features deliver the strongest standalone discriminative power, FD features provide complementary spectral cues, and TF features enhance transient representation, improving robustness under non-stationary disturbances.

#### 5.4.2. Feature Fusion and Ablation Study

To investigate the effectiveness of the multi-domain fusion mechanism, an ablation study is conducted by progressively introducing feature categories. Seven experimental groups are constructed:

- Exp.1 (Baseline): Time features only;
- Exp.2: Frequency features only;
- Exp.3: TF features only;
- Exp.4: Time + Frequency;
- Exp.5: Time + TF;
- Exp.6: Frequency + TF;
- Exp.7 (Proposed): All fused features.

All experiments are conducted on data collected from five railway lines to ensure robustness. Each feature set is normalized using Z-score normalization, and a BiLSTM backbone with Bayesian inference is employed across all comparisons to maintain fairness. Performance is evaluated using three metrics: Acc, the state classification accuracy; MAE, the mean absolute error of capacitance estimation; and ECE, the ECE that measures uncertainty reliability.

As summarized in Table 3, the comparative analysis of different feature configurations reveals clear performance differences across single-domain, dual-domain, and full-fusion strategies. Specifically, single-domain features (Exp. 1–3) show distinct effectiveness characteristics: TD features provide a solid baseline but limited regression precision, whereas FD and TF features yield higher accuracy and stability by capturing spectral and transient characteristics more sensitively. Two-domain fusion (Exp. 4–6) further enhances performance, with all mixed-feature configurations achieving higher accuracy (>95.7%) and lower MAE (<0.12 μF) than their single-domain counterparts. Finally, full multi-domain fusion (Exp. 7) delivers the best results, increasing accuracy to 97.3%, reducing MAE to 0.094 μF, and lowering the Expected Calibration Error (ECE) to 3.9%, reflecting well-calibrated uncertainty estimates.

This demonstrates that multi-domain fusion effectively leverages the complementary strengths of different feature modalities, enabling high robustness in both classification and regression tasks under noisy field conditions.

### 5.5. Recognition and Estimation Performance

#### 5.5.1. State Recognition Performance

A comprehensive evaluation was conducted to assess the effectiveness of the proposed hierarchical Bayesian BiLSTM framework for compensation capacitor state recognition. Real-world inspection data collected from five railway lines were used, covering three capacitor states—Normal, Degraded (Capacitance Drop), and Disconnected (Open Circuit).

After aggregating the section-level data from all lines, the complete dataset was randomly divided into training, validation, and test sets according to a 7:1:2 ratio. The training set was used for model learning, the validation set for hyperparameter tuning and model selection, and the test set was strictly reserved for independent performance evaluation, ensuring reliable generalization assessment.

All compared models employed the same multi-domain feature inputs described in Section 4.2, ensuring a fair comparison. Only the proposed method incorporated Bayesian weight inference based on MC Dropout and SWAG to explicitly model both epistemic and aleatoric uncertainty.

To reduce the influence of random initialization and data partitioning, each experiment was repeated ten times with different random seeds, and the mean performance metrics were reported. The comparison methods included:

- Threshold baseline: rule-based peak/envelope classification;
- Conventional DNN: three-layer MLP without temporal modeling;
- BiLSTM (non-Bayesian): sequence modeling without uncertainty;
- Proposed Bayesian BiLSTM: full feature fusion with hierarchical Bayesian inference.

**Figure 7:**
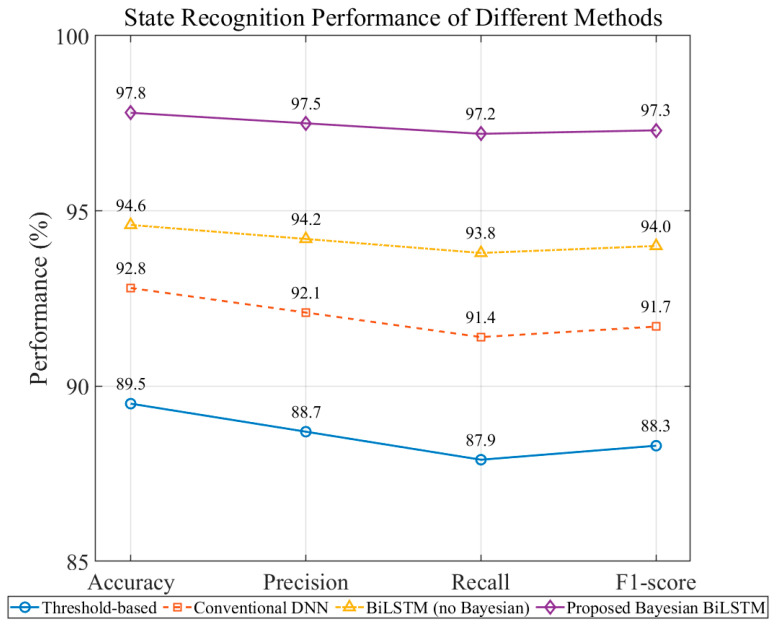
Comparison of Classification Performance in Compensation Capacitor State Recognition Tasks.

As shown in Figure 7, different recognition methods exhibit notable performance differences under complex noise. The traditional threshold method, highly sensitive to noise, drift, and interference, achieves < 90% accuracy with imbalanced recall across states. Conventional DNN and BiLSTM improve accuracy to 92.8% and 94.6%, but their lack of uncertainty modeling leaves them vulnerable to misclassification under distribution shifts or weak signals.

The Proposed Bayesian BiLSTM achieves the best results across all four metrics, reaching 97.8% accuracy—8.3% higher than the threshold baseline and 3.2% higher than the non-Bayesian BiLSTM—with Precision, Recall, and F1-score all exceeding 97%. These improvements stem from: (i) multi-domain enhancement and feature fusion that increase feature separability; (ii) BiLSTM’s ability to capture the capacitor’s entering–centering–leaving dynamics; and (iii) Bayesian inference that mitigates overconfidence in anomalous samples. Overall, the method demonstrates superior robustness under complex conditions, providing a reliable foundation for capacitance estimation and uncertainty analysis.

#### 5.5.2. Capacitance Estimation Performance

To systematically evaluate the proposed Bayesian BiLSTM framework for capacitance estimation, a regression dataset was constructed using signals from five railway lines, comprising 18,000 samples. Each sample contains multi-domain enhanced signal segments and corresponding labeled capacitance values spanning normal ranges (9.5–10.5 μF), mildly degraded ranges (8–9.5 μF), and severely degraded or near–open-circuit states (<8 μF), ensuring that model performance is assessed across the full operational lifecycle. Comparative baselines included a threshold-based method that performs coarse estimation using peak or energy criteria, a two-layer fully connected DNN, and a non-Bayesian BiLSTM that provides sequence modeling without uncertainty estimation. The proposed Bayesian BiLSTM integrates multi-domain features, temporal modeling, and Bayesian inference. Evaluation was performed using MAE, RMSE, R^2^, and the width of the 95% confidence interval to quantify both accuracy and prediction reliability.

**Figure 8:**
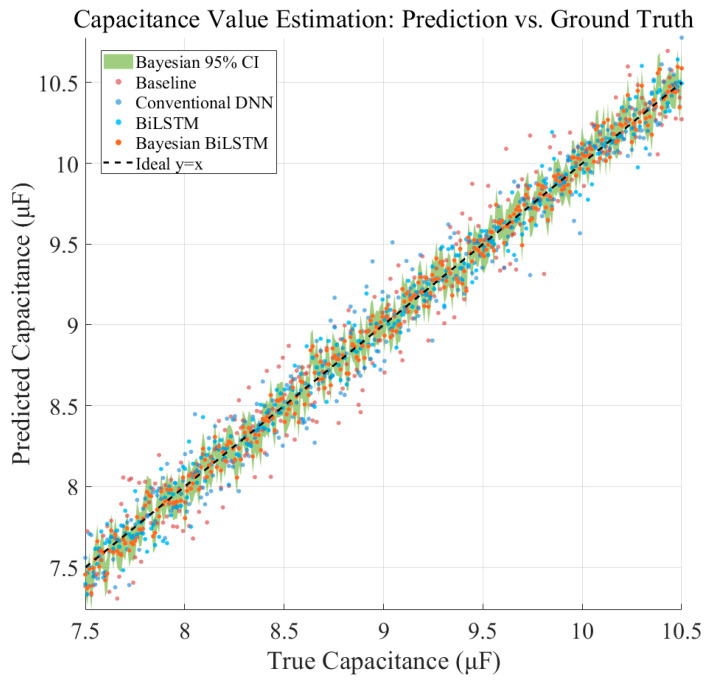
Predicted versus Reference Capacitance for Different Estimation Methods.

As shown in Table 4 and Figure 8, the threshold-based method yields the largest estimation errors (MAE = 0.18 μF) due to its fixed-rule and noise-sensitive nature. Conventional deep neural networks reduce the estimation error but remain unstable under varying operating conditions (R^2^ = 0.87). By explicitly modeling temporal dependencies, the BiLSTM further improves estimation performance, reducing MAE to 0.119 μF.

The proposed Bayesian BiLSTM achieves the best overall performance, with MAE = 0.084 μF, RMSE = 0.119 μF, and R^2^ = 0.96, while additionally providing a reliable 95% confidence interval with an average width of 0.21 μF. These results demonstrate that the proposed framework not only improves capacitance estimation accuracy but also effectively characterizes predictive uncertainty through Bayesian posterior sampling, making it well suited for safety-critical railway track circuit maintenance.

#### 5.5.3. Uncertainty Modeling and Confidence Analysis

This section evaluates the reliability of the proposed Bayesian framework through predictive uncertainty estimation and confidence-based rejection. Traditional deterministic models offer only point predictions and cannot assess reliability under noise, anomalies, or distribution shifts. In contrast, the proposed model jointly models aleatoric and epistemic uncertainty, outputs predictive distributions and confidence intervals, and incorporates a rejection mechanism to avoid low-confidence decisions.

Following the same protocol as Section 5.5.1 and Section 5.5.2, the Baseline, non-Bayesian BiLSTM, and Proposed Bayesian BiLSTM are compared using real-world detection data from the five lines. The evaluation metrics include: (i) confidence interval coverage, assessing whether true values fall within predicted intervals; (ii) predictive variance, reflecting distribution-level uncertainty; and (iii) rejection rate (RR), measuring the proportion of predictions excluded for low confidence. All models are trained and tested with identical data and feature inputs to ensure fair and reproducible comparisons.

**Figure 9:**
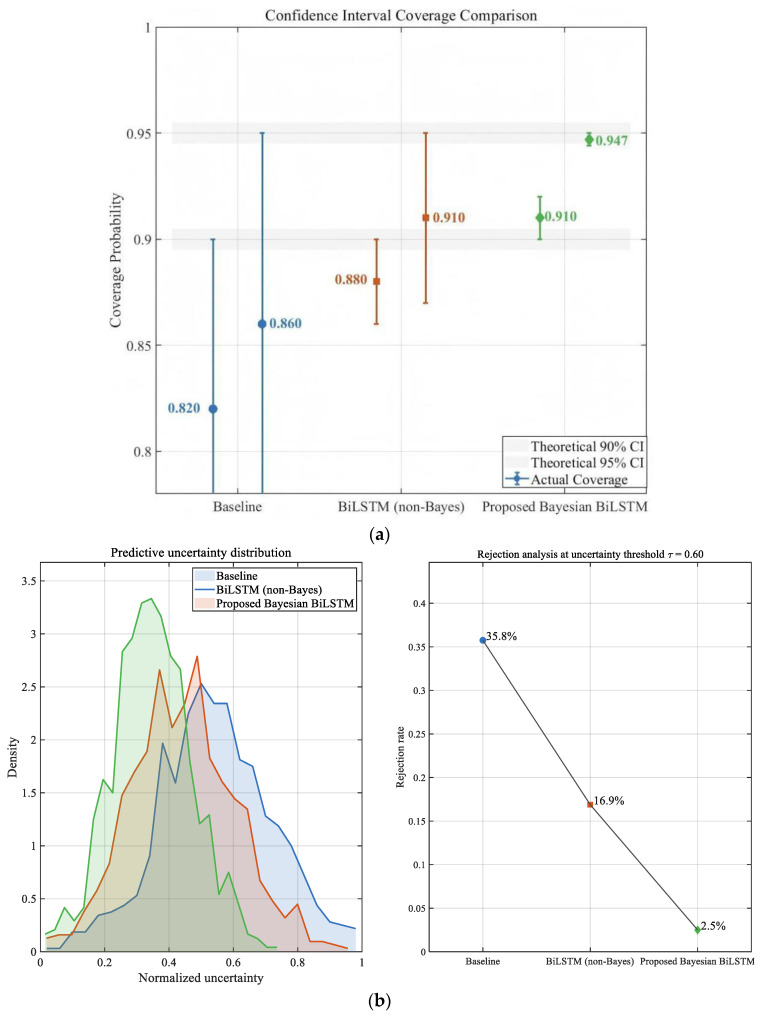
Uncertainty Calibration and Confidence-Based Reliability Analysis. (**a**) Confidence Interval Coverage Comparison. (**b**) Uncertainty Distribution and Rejection Rate Analysis.

Figure 9a compares the empirical coverage rates of different methods at the 90% and 95% confidence intervals. The Baseline and non-Bayesian models exhibit clear under-calibration, with actual 95% CI coverage reaching only 0.86–0.91, indicating that their predictive intervals are too narrow to capture uncertainty arising from operational variations. In contrast, the proposed Bayesian BiLSTM achieves the highest calibration performance, reaching 94.7% coverage at the 95% CI—close to the theoretical value—and its error bars display a more concentrated and stable uncertainty distribution, suggesting stronger robustness to noise fluctuations.

Here, the normalized uncertainty refers to a dimensionless predictive uncertainty indicator obtained by normalizing the model-estimated variance with respect to the corresponding reference capacitance and is used for comparative analysis and confidence-based rejection rather than for expressing uncertainty in physical units. Figure 9b evaluates predictive variance and rejection behavior. Baseline and conventional networks show large, noise-sensitive predictive variances and lack effective rejection mechanisms. The non-Bayesian BiLSTM reduces variance through sequential feature modeling but still produces a considerable number of low-confidence samples. The proposed Bayesian BiLSTM attains the lowest average predictive variance and the most effective rejection performance: for samples influenced by strong noise or abrupt condition changes, the model identifies high predictive uncertainty and activates rejection at a rate of approximately 6–8%. Its variance distribution demonstrates a weakened right-skewed tail, indicating enhanced risk-awareness and the capability to recognize low-reliability predictions.

Overall, the proposed method achieves superior uncertainty modeling, calibration performance, and rejection capability.

## 6. Discussion and Conclusions

This work introduces a hierarchical Bayesian deep learning framework that unifies multi-domain signal enhancement, feature fusion, sequential modeling, and uncertainty-aware inference for compensation capacitor detection in track circuits. By jointly addressing state recognition, capacitance regression, and predictive uncertainty, the framework overcomes the limited robustness, rule dependence, and lack of credibility estimation inherent in traditional threshold-based methods. Multi-domain enhancement effectively suppresses noise, drift, and narrowband interference, while BiLSTM captures the characteristic temporal evolution of the capacitor’s entering–centering–leaving process. Bayesian inference with MC Dropout and SWAG produces calibrated confidence intervals and provides risk-aware outputs, forming a reliable safety mechanism under complex operating conditions.

From a measurement perspective, it should be emphasized that the proposed uncertainty modeling approach is data-driven rather than analytically metrological. Instead of constructing an explicit measurement uncertainty budget—which would require detailed physical modeling of all contributing uncertainty sources such as antenna positioning, electromagnetic field distribution, rail conditions, and environmental variability—the proposed framework quantifies predictive uncertainty directly from real-world data. As a result, the estimated uncertainty reflects the combined effects of measurement noise, modeling errors, and environmental influences, implicitly captured during training and evaluation on real-world railway data. In this sense, the proposed Bayesian uncertainty estimation can be regarded as a practical, data-driven surrogate representation of classical measurement uncertainty, complementing traditional metrology-oriented analysis in complex railway environments.

Experimental evaluations across multiple real-world railway lines demonstrate that the proposed approach consistently outperforms both threshold-based and conventional deep learning models in recognition accuracy, capacitance estimation precision, and confidence calibration, validating its effectiveness for field deployment.

In addition to its methodological contributions, the framework offers high engineering value by enabling automated detection, improving diagnostic consistency, reducing maintenance cost, and enhancing system-level safety through uncertainty-driven rejection of unreliable predictions. From an implementation perspective, although the proposed framework incorporates Bayesian inference mechanisms, its computational complexity remains compatible with practical deployment scenarios. The core components are based on lightweight one-dimensional convolution and BiLSTM architectures, and Bayesian uncertainty estimation is achieved through stochastic forward passes without introducing additional model parameters. For resource-constrained systems, such as onboard inspection or edge-computing platforms, computational overhead can be further reduced by limiting the number of stochastic samples during inference, adopting model compression or pruning techniques, or selectively enabling uncertainty estimation only for low-confidence samples. These strategies make the proposed approach feasible for practical railway signal monitoring systems with limited computational resources.

Despite its strong performance, challenges remain regarding dependency on labeled data diversity, computational overhead of Bayesian sampling, and robustness under extreme signal perturbations. Future research will explore unsupervised and semi-supervised learning strategies to reduce reliance on labeled data, long-term sensor degradation modeling for predictive maintenance, transfer learning and data augmentation to improve generalization, lightweight Bayesian architectures for real-time deployment, and multimodal data fusion to enhance robustness under complex and abnormal operating conditions. Overall, the proposed method provides a robust, trustworthy foundation for intelligent track circuit maintenance and represents a step toward reliable, uncertainty-aware AI systems in railway signal engineering.

## Figures and Tables

**Table 1:** Dataset Size and Class Distribution.

Line ID	No. of Segments	Normal Capacitors (70%)	Degraded Capacitance Drop (20%)	Disconnected Open Circuit (10%)	Total Samples
Line 1	2334	1634	467	233	2334
Line 2	1875	1313	375	187	1875
Line 3	2034	1424	407	203	2034
Line 4	894	626	179	89	894
Line 5	1645	1151	329	165	1645
Overall	8782	6148	1757	877	8782

**Table 2:** Evaluation Metrics.

Metric Category	Metric	Definition and Description	Evaluation Purpose
Classification	Accuracy	Ratio of correctly classified samples to all samples.	Overall ability to distinguish the three capacitor states (Normal/Degraded/Disconnected).
	Precision	TP/(TP+FP); proportion of correct predictions within the predicted class.	Measures the reliability of fault predictions.
	Recall	TP/(TP+FN); proportion of samples of a given class that are correctly detected.	Reflects the missed-detection rate for critical faults.
	F1-score	Harmonic mean of precision and recall.	Balanced evaluation of fault-classification performance.
Regression	MAE	Mean absolute deviation between predicted and true capacitance values.	Measures overall estimation bias.
	RMSE	Square root of mean squared error. More sensitive to large errors.	Evaluates estimation stability under extreme or noisy conditions.
Uncertainty and Reliability	NLL	Evaluates probabilistic prediction quality by assessing the likelihood of ground truth under the predicted probability distribution, reflecting both prediction accuracy and uncertainty estimation.	Evaluates calibration quality of probabilistic outputs.
	ECE	Evaluates confidence calibration by comparing predicted confidence levels with empirical accuracy across confidence intervals.	Assesses the reliability of predicted probabilities.
	RR	Rejection Rate; fraction of samples rejected by the confidence-based mechanism.	Evaluates the system’s ability to filter high-risk or unreliable predictions.

**Table 3:** Experimental results under different feature fusion strategies.

Experiment	Feature Type	Acc (%)	MAE (μF)	ECE (%)
Exp.1	Time only	91.2	0.158	7.3
Exp.2	Frequency only	92.5	0.141	6.9
Exp.3	TF only	93.8	0.132	6.4
Exp.4	Time + Frequency	95.1	0.118	5.2
Exp.5	Time + Time–Freq.	95.4	0.115	5.0
Exp.6	Freq. + Time–Freq.	95.7	0.112	4.8
Exp.7 (Proposed)	All fused	97.3	0.094	3.9

**Table 4:** Comparison of Capacitance Estimation Performance.

Method	MAE (μF)	RMSE (μF)	R^2^	95% CI Width (μF)
Threshold-based [Baseline]	0.182	0.226	0.83	-
Conventional DNN	0.141	0.187	0.87	-
BiLSTM (no Bayesian)	0.117	0.151	0.91	-
Proposed Bayesian BiLSTM	0.084	0.119	0.96	0.21

## Data Availability

Data are contained within the article.

## References

[1] de Bruin T., Verbert K., Babuška R. (2017). Railway Track Circuit Fault Diagnosis Using Recurrent Neural Networks. IEEE Trans. Neural Netw. Learn. Syst..

[2] Peng F., Liu T. (2024). Method for fault diagnosis of track circuits based on a TF intelligent network. Electronics.

[3] Tao W., Li X., Li Z. (2024). Track circuits fault diagnosis method based on the UNet-LSTM network (ULN). J. Electr. Comput. Eng..

[4] Shang Y., Yan F., Feng S., Wang Z. (2021). Fault diagnosis of compensation capacitor in joint-less track circuit via long short-term memory network. Proceedings of the 2021 China Automation Congress (CAC).

[5] Song H., Li L., Li Y., Tan L., Dong H. (2024). Functional Safety and Performance Analysis of Autonomous Route Management for Autonomous Train Control System. IEEE Trans. Intell. Transp. Syst..

[6] Song H., Gao S., Li Y., Liu L., Dong H. (2023). Train-centric communication based autonomous train control system. IEEE Trans. Intell. Veh..

[7] Li X., Zhang W., Ding Q., Sun J.-Q. (2020). Intelligent rotating machinery fault diagnosis based on deep learning using data augmentation. J. Intell. Manuf..

[8] Zhang Y., Li X., Gao L., Chen W., Li P. (2020). Intelligent fault diagnosis of rotating machinery using a new ensemble deep auto-encoder method. Measurement.

[9] Gal Y., Ghahramani Z. (2016). Dropout as a bayesian approximation: Representing model uncertainty in deep learning. Proceedings of the International Conference on Machine Learning.

[10] Maddox W.J., Izmailov P., Garipov T., Vetrov P.D., Wilson A.G. A simple baseline for bayesian uncertainty in deep learning. Proceedings of the Annual Conference on Neural Information Processing Systems 2019 (NeurIPS 2019).

[11] Morimoto M., Fukami K., Maulik R., Vinuesa R., Fukagata K. (2022). Assessments of epistemic uncertainty using Gaussian stochastic weight averaging for fluid-flow regression. Phys. D Nonlinear Phenom..

[12] Xie J., Ma Z., Lei J., Zhang G., Xue J.-H., Tan Z.-H., Guo J. (2021). Advanced dropout: A model-free methodology for Bayesian dropout optimization. IEEE Trans. Pattern Anal. Mach. Intell..

[13] Das L., Gjorgiev B., Sansavini G. (2024). Uncertainty-aware deep learning for monitoring and fault diagnosis from synthetic data. Reliab. Eng. Syst. Saf..

[14] Ren J., Wen J., Zhao Z., Yan R., Chen X., Nandi A.K. (2024). Uncertainty-aware deep learning: A promising tool for trustworthy fault diagnosis. IEEE/CAA J. Autom. Sin..

[15] Yao Y., Han T., Yu J., Xie M. (2024). Uncertainty-aware deep learning for reliable health monitoring in safety-critical energy systems. Energy.

[16] Ji A., Woo W.L., Wong E.W.L., Quek Y.T. (2021). Rail track condition monitoring: A review on deep learning approaches. Intell. Robot..

[17] Di Summa M., Griseta M.E., Mosca N., Patruno C., Nitti M., Renò V., Stella E. (2023). A review on deep learning techniques for railway infrastructure monitoring. IEEE Access.

[18] Tang R., De Donato L., Besinović N., Flammini F., Goverde R.M., Lin Z., Liu R., Tang T., Vittorini V., Wang Z. (2022). A literature review of Artificial Intelligence applications in railway systems. Transp. Res. Part C Emerg. Technol..

[19] Oh K., Yoo M., Jin N., Ko J., Seo J., Joo H., Ko M. (2022). A review of deep learning applications for railway safety. Appl. Sci..

[20] Tao W., Li X., Liu J., Li Z. (2024). Multi-scale attention network (MSAN) for track circuits fault diagnosis. Sci. Rep..

[21] Na L., Cai B., Zhang C., Liu J., Li Z. (2025). A heterogeneous transfer learning method for fault prediction of railway track circuit. Eng. Appl. Artif. Intell..

[22] Li Y., Rao Z., Li Z., Ding L. (2022). Research on Fault Location Method of Track Circuit Compensation Capacitor Based on Probabilistic Neural Network. Archit. Eng. Sci..

[23] Gao H., Shi J., Bao C., Li P., Chen G. (2023). Fault prediction of track circuit compensation capacitor based on MFO-LSTM. Proceedings of the 2023 CAA Symposium on Fault Detection, Supervision and Safety for Technical Processes (SAFEPROCESS).

[24] Wang C., Yang S., Liu C. (2024). A novel health state prediction approach based on artificial intelligence combination strategy for compensation capacitors in track circuit. J. Supercomput..

[25] Chen G., Wang S., Li P., Zhou X., Zhao S., Shi J., Bao C. (2025). A phase space and network-based approach for diagnosing compensation capacitor faults in Jointless Track Circuits. Measurement.

[26] Cheliotis M., Lazakis I., Cheliotis A. (2022). Bayesian and machine learning-based fault detection and diagnostics for marine applications. Ships Offshore Struct..

[27] Zhao J., Wang W., Huang J., Ma X. (2025). A comprehensive review of deep learning-based fault diagnosis approaches for rolling bearings: Advancements and challenges. AIP Adv..

[28] Zhou T., Han T., Droguett E.L. (2022). Towards trustworthy machine fault diagnosis: A probabilistic Bayesian deep learning framework. Reliab. Eng. Syst. Saf..

[29] Zhou T., Zhang L., Han T., Droguett E.L., Mosleh A., Chan F.T. (2023). An uncertainty-informed framework for trustworthy fault diagnosis in safety-critical applications. Reliab. Eng. Syst. Saf..

[30] Meng L., Su Y., Kong X., Lan X., Li Y., Xu T., Ma J. (2022). A probabilistic bayesian parallel deep learning framework for wind turbine bearing fault diagnosis. Sensors.

[31] Jalayer R., Jalayer M., Mor A., Orsenigo C., Vercellis C. (2024). Evaluating deep learning models for fault diagnosis of a rotating machinery with epistemic and aleatoric uncertainty. arXiv.

[32] Lin Y.H., Li G.H. (2024). Uncertainty-aware fault diagnosis under calibration. IEEE Transactions on Systems, Man, and Cybernetics: Systems.

[33] Li H., Jiao J., Liu Z., Lin J., Zhang T., Liu H. (2025). Trustworthy Bayesian deep learning framework for uncertainty quantification and confidence calibration: Application in machinery fault diagnosis. Reliab. Eng. Syst. Saf..

[34] Mostafavi A., Siami M., Friedmann A., Barszcz T., Zimroz R. Probabilistic Uncertainty-Aware Decision Fusion of Neural Network for Bearing Fault Diagnosis. Proceedings of the Prognostics and Health Management Society (PHM European Conference) 2024.

